# Biological Effects of Phosphocitrate on Osteoarthritic Articular Chondrocytes

**DOI:** 10.2174/1874312901711010062

**Published:** 2017-05-31

**Authors:** Yubo Sun, Atiya M Franklin, David R Mauerhan, Edward N Hanley

**Affiliations:** Department of Orthopedic Surgery, Cannon Research, Carolinas Medical Center, PO Box 32861, Charlotte, NC 28232, USA

**Keywords:** Chondrocyte, Calcification, Matrix, Microarray, Osteoarthritis, Phosphocitrate

## Abstract

**Background:**

Phosphocitrate (PC) inhibits osteoarthritis (OA) in Hartley guinea pigs. However, the underlying molecular mechanisms remain poorly understood.

**Objective:**

This study sought to examine the biological effect of PC on OA chondrocytes and test the hypothesis that PC may exert its OA disease modifying effect, in part, by inhibiting the expression of genes implicated in OA disease process and stimulating the production of extracellular matrices.

**Method:**

OA chondrocytes were cultured in the absence or presence of PC. Total RNA was extracted and subjected to microarray analyses. The effect of PC on proliferation and chondrocyte-mediated calcification were examined in monolayer culture. The effect of PC on the production of extracellular matrices was examined in micromass culture.

**Results:**

PC downregulated the expression of numerous genes classified in proliferation and apoptosis while upregulating the expression of many genes classified in transforming growth factor-β (TGF-β) receptor signaling pathway and ossification. PC also downregulated the expressions of many genes classified in inflammatory response and Wnt receptor signaling pathways. Consistent with its effect on the expression of genes classified in proliferation, ossification, and skeletal development, PC inhibited the proliferation of OA chondrocytes and chondrocyte-mediated calcification while stimulating the production of extracellular matrices.

**Conclusion:**

PC may exert its OA disease modifying effect, in part, through a crystal-independent mechanism or by inhibiting the expressions of many genes implicated in OA disease process, and at the same time, stimulating the expression of genes implicated in chondroprotection and production of extracellular matrices.

## 1 INTRODUCTION

Osteoarthritis (OA) is a degenerative joint disease characterized by gradual loss of articular cartilage, formation of osteophytes, and synovial inflammation. Existing pharmacological interventions for OA remain insufficient. Widely prescribed non-steroid anti-inflammatory drugs and steroid knee injections only relieve pain and inflammation but have no effects on the progression of cartilage degeneration. There is a need for the development of structural disease-modifying drugs that not only relieve pain and inflammation but also inhibit or halt the progression of cartilage degeneration. The lack of progress in the development of structural disease-modifying drugs is largely due to our limited understanding of the pathogenesis of OA and insufficient knowledge regarding the molecular targets for therapeutic intervention.

The biochemical events involved in the initiation and progression of OA are poorly understood. Extracellular matrix degrading enzymes and inflammatory cytokines, including matrix metalloproteinases (MMPs), ADAM metallopeptidase with thrombospondin type 1 motif 5 (ADAMTS5), interleukin-1 (IL-1), and tumor necrosis factor alpha (TNF-α) have been implicated in OA. Small molecules and biologics targeting MMPs, IL-1, and TNF-α have been examined for OA intervention, however, results of clinical trials with MMP inhibitor(s) and biologics against IL-1 and TNF-α were disappointing [1-4]. Pathological calcification has also been implicated in OA. Basic calcium phosphate (BCP) crystals and calcium pyrophosphate dihydrate (CPPD) crystals are present in the synovial fluid, menisci, and articular cartilage of patients with end-stage OA [5-7]. These crystals stimulated mitogenesis and the production of MMPs and inflammatory cytokines in cell cultures [8-11] and induced a severe inflammatory response within the joints of dogs and mice [12, 13].

Phosphocitrate (PC) is a powerful calcification inhibitor, inhibiting calcification or formation of calcium crystals by binding to amorphous calcium phosphate aggregates and the surface of calcium crystals. PC inhibited BCP crystal-induced mitogenesis, expression of MMPs, and cell death [14-16]. Based on these findings, a hypothesis that PC is potentially a disease-modifying drug for calcification-induced OA therapy was postulated [17]. A subsequent study demonstrated that PC inhibited meniscal calcification and that the reduction in meniscal calcification was accompanied with decreased cartilage degeneration in Hartley guinea pigs [18]. It was believed that PC exerted its disease modifying activity by inhibiting the formation of calcium crystals and the detrimental interactions between these crystals and joint cells (a crystal-dependent mechanism) [17].

We recently demonstrated that PC downregulated the expression of many genes classified in cell proliferation, angiogenesis, and inflammatory response in the absence of calcium crystals in OA fibroblast-like synoviocytes (FLSs) and OA meniscal cells [19-21]. These newer findings suggest that PC exerts its OA disease-modifying activity, in part, through a crystal-independent mechanism, directly acting on OA cells instead of acting on calcium crystals. Indeed, PC inhibited cartilage degeneration in Hartley guinea pig model of posttraumatic OA through both a crystal-dependent mechanism and a crystal-independent mechanism [22]. However, the molecular mechanisms remain poorly understood. In this study, we sought to examine the biological effects of PC on OA articular chondrocytes and test the hypothesis that PC may exert its OA disease modifying effect, in part, by inhibiting the expression of genes implicated in OA disease process and stimulating the production of extracellular matrices. The information gained from this study may not only shed light on the molecular mechanism underlying the OA disease-modifying activity of PC and provide information valuable for the identification of potential disease candidate genes but also may provide information valuable for a better understanding of the pathogenesis of OA.

## 2 MATERIALS AND METHODS

Dulbecco’s modified eagle medium, StemPro osteogenesis differentiation medium, StemPro chondrogenesis differentiation medium, fetal bovine serum, Hank’s balanced salt solution, antibiotic and antimycotic solution were products of Invitrogen (Carlsbad, CA). Human foreskin fibroblasts were obtained from American Type Culture Collection (CRL-2429, Manassas, VA). PC was synthesized according to the procedures described [23]. In all experiments, 1mm PC was used because it has been used in previous studies and shows no toxic effect on cells [15, 19].

### 2.1 OA Articular Chondrocytes

OA chondrocytes were prepared from articular cartilage specimens collected from 6 end-stage OA patients (age range of 62-68) undergoing joint replacement surgery at Carolinas Medical Center with the approval of the authors’ Institutional Review Board. The need for informed consent was waived because those specimens were surgical waste, and no private patient information was collected. Articular cartilage was evaluated as described [24], and only grade 4 cartilages were used. Briefly, articular cartilage was removed from the medial tibia plateau with a surgical blade, minced into small pieces, and cultured in 60 mm plates at 37^o^C in medium containing 10% serum and 0.5% antibiotic/antimycotic solution. Media was changed every 3 days. After chondrocytes reached to 70% confluence, they were passaged. Chondrocytes with 2-3 passages were used in the study.

### 2.2 Effect of PC on Gene Expressions

OA chondrocytes derived from 3 OA patients were harvested from 60 mm cell culture plates, mixed, and re-plated in 60 mm plates at 90% confluence (passage 2; 9 x 10^6^ per plate). On the second day, medium containing 1% serum was added. On the third day, medium in 2 plates was replaced with medium containing 1% serum and 1 mm PC and medium in the other 2 plates were replaced with medium containing 1% serum without PC as a control. Twenty-four hours later, total RNA was extracted using Trizol reagent (Invitrogen, Carlsbad, CA) and purified using the Oligotex kit (Qiagen, Valencia, CA). The quality and integrity of these RNA samples were checked using RNA 6000 Nano Assay Reagent Kit and Agilent 2100 Bioanalyzer (Agilent Technologies, Santa Clara, CA).

RNA samples extracted from two independent experiments were used for microarray analyses. Briefly, double-stranded DNA was synthesized using SuperScript Double-Stranded cDNA Synthesis Kit (Invitrogen, San Diego, CA, USA) using 1 μg of each RNA sample. The DNA product was purified using a GeneChip sample cleanup module (Affymetrix, Santa Clara, CA, USA). CRNA was synthesized and biotin labeled using BioArray high yield RNA transcript labeling kit (Enzo Life Sciences, Farmingdale, NY, USA). The cRNA product was purified using GeneChip sample cleanup module and subsequently chemically fragmented. The fragmented and biotinylated cRNA was hybridized to HG-U133_Plus_2.0 gene chip using Affymetrix Fluidics Station 400 (Affymetrix, Santa Clara, CA, USA). The fluorescent signals were quantified during two scans by Agilent Gene Array Scanner G2500A (Agilent Technologies, Palo Alto, CA, USA) and GeneChip operating Software (Affymetrix, Santa Clara, CA, USA). Genesifter (VizX Labs, Seattle, WA, USA) was used for the analysis of differential gene expression and gene ontology. The results of the two microarray experiments were similar, therefore, we focused on the results of the first microarray experiment in this study.

### 2.3 Real-time RT-PCR

The RNA samples used in microarray experiments were used for real-time RT-PCR. Briefly, cDNA was synthesized using TaqMan^®^ Reverse Transcription Reagents (Applied Biosystems, University Park, IL) using 1 μg RNA sample. Quantification of relative transcript levels of selected genes and the housekeeping gene glyceraldehyde 3-phosphate dehydrogenase (GAPDH) was performed using TaqMan^®^ Gene Expression assay and ABI7000 Real-Time PCR system (Applied Biosystems, University Park, IL). CDNA samples were amplified with an initial Taq DNA polymerase activation step at 95^o^C for 10 minutes, followed by 40 cycles of denaturation at 95^o^C for 15 seconds and annealing at 60^o^C for one minute. Fold change was calculated and the expression level of a specific gene was normalized to the expression level of GAPDH.

### 2.4 Effect of PC on the Proliferation of OA Chondrocytes

OA chondrocytes (2x10^4^) were plated in six-well cluster plates and cultured in medium containing 10% serum in the absence (triplicates) or the presence of 1 mM PC (triplets). The medium was changed every three days until the chondrocytes in the wells without PC reached to 85% confluence (it took about 14-16 days). Chondrocytes in all wells were harvested and cell numbers counted. This experiment was repeated three times using OA chondrocytes derived from 3 different OA patients. Trypan blue test was performed to examine the effect of PC on chondrocyte viability. Since foreskin fibroblasts were used in many previous studies to determine the effect of PC on crystal-induced mitogenesis and expression of MMPs [15, 25, 26], foreskin fibroblasts were also used here for comparison.

### 2.5 Effect of PC on Chondrocyte-Mediated Calcification

OA chondrocytes were plated in twenty-four well cluster plates at 90% confluence (7 x 10^5^ cells). On the second day, the medium was replaced with StemPro osteogenesis differentiation medium with or without 1 mm PC (triplicates). These chondrocytes were fed with StemPro osteogenesis differentiation medium with or without PC every three days. Fourteen days later, the media were removed and calcium deposition was examined using alizarin red. This experiment was repeated 6 times using OA chondrocytes derived from 6 different OA patients.

### 2.6 Effect of PC on the Production of Extracellular Matrices in Micromass Culture

Micromasses were prepared as described previously [7, 27]. Briefly, OA chondrocytes were harvested from 60 mm culture plates and suspended in DMEM containing 10% serum. For preparing a micromass, a droplet of the cell suspension containing 2 x 10^5^ chondrocytes was placed in a well of a 24-well cluster plate. After placing all droplets, the plate was incubated for 4 hours at 37^0^C in a tissue culture incubator and each of the micromasses formed was then fed with StemPro chondrogenesis differentiation medium. Starting on the next day, these micromasses were fed with StemPro chondrogenesis differentiation medium with PC (1 mM) or without PC every three days.

Fourteen days later each well was rinsed twice with 500 μl of Hank’s balanced salt solution and two drops of eosin were added. Five minutes later, eosin was aspirated off and the micromasses were transferred to a strip of filter paper which sat on the top of an ethanol-soaked sponge within a plastic cassette. The cassettes sat in a 10% formalin solution for one hour. These micromasses then underwent routine paraffin embedding. Sections were cut at 5 μm thick and stained with picrosirius red for collagens and alcian blue for proteoglycans. This experiment was repeated 3 times using OA chondrocytes derived from 3 different patients.

### 2.7 Statistical Analysis

Results are expressed as the mean ± standard error. The difference between the PC-treated and untreated group was analyzed using Student's t-test. It was considered significant when the p-value was < 0.01. Statistical analysis was performed using the statistical analysis tool in the Sigma Plot software, version 12 (Systat software Inc., San Jose, CA).

## 3 RESULTS

### 3.1 Effect of PC on Gene Expressions

Of more than 50,000 transcripts, 558 transcripts displayed differential expression (more than 1.5 fold changes) in PC-treated OA chondrocytes compared to untreated OA chondrocytes. A total of 334 transcripts displayed decreased expression and 224 transcripts displayed increased expression. The genes that fell into specific biological processes implicated in OA or suspected to have a role in OA are listed in (Tables **1** and **2**).

As shown in Table **1**, the expression of numerous genes classified in proliferation and apoptosis was down-regulated by PC. Of the 39 differentially-expressed genes classified in cell proliferation, the expression of 30 genes, including lymphoid-specific helicase (HELLS, -2.32 fold) and interleukin 7 (IL-7, -1.82 fold), was down-regulated by PC. Of the 38 differentially-expressed genes classified in apoptosis, the expression of 30 genes, including sprouty homolog 2 (SPRY2, -1.92 fold) and baculoviral IAP repeat-containing 5 (BIRC5, -1.76 fold), was down-regulated by PC. In contrast, the expression of many genes classified in the transforming growth factor β (TGF-β) receptor signaling pathway and ossification were upregulated by PC. Of the 5 differentially-expressed genes classified in the TGF-β receptor signaling pathway, the expression of all 5 genes, including TGF-beta receptor 1 (TGFBR1, 1.74 fold), SMAD specific E3 ubiquitin protein ligase 1 (SMURF1, 1.57 fold), and SMAD family member 3 (SMAD3, 1.55 fold), was up-regulated by PC. Of the 7 differentially-expressed genes classified in ossification, the expression of 5 genes, including fibroblast growth factor receptor 2 (FGFR2, 1.99 fold), PDZ and LIM domain 7 (PDLIM7, 1.57 fold), and insulin-like growth factor binding protein 5 (IFGBP5, 1.53 fold), was up-regulated by PC. However, ectonucleotide pyrophosphatase 1 (ENPP1, -1.66 fold) and bone morphogenetic protein 2 (BMP2, -1.55 fold), two genes implicated in promoting ossification, was down-regulated by PC.

The expression of many genes classified in the inflammatory response, skeletal system development, and Wnt receptor signaling pathway was also downregulated by PC (Table **2**). Of the 6 differentially-expressed genes classified in the inflammatory response, the expression of 4 genes, including fatty acid-binding protein 4 (FABP4; -2.11 fold), interleukin-1 receptor-associated kinase 2 (IRAK2, -1.71), and interleukin 1 receptor accessory protein (IL1RAP, -1.57), were down-regulated by PC of the 8 differentially-expressed genes classified in skeletal system development, the expression of 5 genes, including distal-less homeobox 2 (DLX2, -2.37 fold) and periostin (POSTN, -1.56 fold) was down-regulated by PC, of the 8 differentially-expressed genes classified in the Wnt receptor signaling pathway, the expression of 6 genes, including adenomatosis polyposis coli down-regulated 1 (APCDD1, -2.68 fold), norrie disease (NDP, -2.30 fold), and frizzled homolog 8 (FZD8, -1.58 fold), was down-regulated by PC.

### 3.2 Real-Time RT-PCR

Real-time RT-PCR was performed to confirm the differential expression of selected genes. As shown in Table **3**, the differential expression of the genes examined was confirmed by real-time RT-PCR (P < 0.01).

### 3.3 PC Inhibits Proliferation of OA Chondrocytes

The down-regulatory effect of PC on the expression of numerous genes classified in cell proliferation suggests that PC inhibits the proliferation of OA chondrocytes. Indeed, there were roughly 45% fewer OA chondrocytes in the wells containing PC compared to the wells containing no PC Fig. (**1**). The morphology of OA chondrocytes in the wells with and without PC was similar and trypan blue test showed little dead cells in both the untreated and PC-treated chondrocytes (not shown).

### 3.4 PC Inhibits OA Chondrocyte-Mediated Calcification

To investigate the OA chondrocyte-mediated calcification, we cultured OA chondrocytes in osteogenetic differentiation medium. As shown in Fig. (**2**), calcium deposits were detected in the monolayer of OA chondrocytes cultured in osteogenetic differentiation medium in the absence of PC, but not in the presence of 1 mm PC. These findings demonstrated that OA chondrocytes were capable of producing calcium crystals and that PC inhibited this calcification process.

### 3.5 PC Stimulates the Production of Cartilaginous Extracellular Matrices

Representative images of untreated and PC-treated micromasses of OA chondrocytes are provided in Fig. (**3**). As shown, the intensity of picrosirius red staining in the outer layer of the PC-treated micromass of OA chondrocytes was higher than the intensity of picrosirius red staining in the outer layer of the untreated micromass, indicating that PC stimulated the production of collagens by OA chondrocytes. The intensity of alcian blue staining in the outer layer of the PC-treated micromass of OA chondrocytes was also higher than the intensity of alcian blue staining in the untreated micromass, indicating that PC also stimulated the production of proteoglycans by OA chondrocytes.

## 5 DISCUSSION

Increased number and size of chondrocyte clusters are hallmark histological features of OA articular cartilage [28, 29]. The number of proliferating chondrocytes increased during OA progression [29, 30] and the clusters contained apoptotic chondrocytes [29-32]. One of the potential mechanisms of apoptosis is abnormal differentiation of OA chondrocytes and subsequent calcification [33, 34]. OA cartilage showed co-localization of chondrocyte clusters and calcium deposits adjacent to apoptotic chondrocytes [35]. These previous findings indicate that chondrocyte activation, differentiation, calcification, and apoptosis are mutually linked OA disease processes. In this study, we demonstrate that PC downregulated the expression of numerous genes classified in proliferation and apoptosis in OA chondrocyte culture in the absence of calcium crystals. Consistent with its gene expression-regulatory effects, PC inhibited the proliferation of OA chondrocytes and chondrocyte-mediated calcification. These findings together indicate that PC can act directly on OA chondrocytes (a crystal-independent action of PC) in addition to calcium crystals (a crystal-dependent action of PC). PC may exert its OA disease-modifying activity, in part, by targeting genes involved in chondrocyte activation, apoptosis, and calcification, and in so doing, inhibiting chondrocyte apoptosis and chondrocyte-mediated calcification [36, 37].

Several previous findings appeared to be consistent with this new molecular mechanism. For examples, IL-7 (classified in proliferation) level was higher in OA chondrocytes and stimulated the production of MMP-13 [38]; HELLS (classified in proliferation and apoptosis) promoted cell activation [39]; BIRC5 (classified in apoptosis) was observed in chondrocytes within degraded lesions and was positively associated with apoptotic chondrocytes [40]. In this study, we demonstrated that PC down-regulated the expression of these 3 genes: IL-7, HELLS, and BIRC5. In addition, a recent study demonstrated that signaling via TGFBR1 (Smad2/3 route) resulted in a protective response whereas signaling via activin A receptor type II-like 1 (ACVRL1) (Smad1/5/8 route) resulted in a deleterious response in articular chondrocytes [41]. The ratio of ACVRL1/TGFBR1 was significantly increased in the cartilage of aging mice, favoring TGF-β signaling via the Smad1/5/8 route, inducing changes in chondrocyte differentiation and MMP-13 expression [41]. These previous findings indicate that reduced signaling via TGFBR1 (Smad2/3 route) may play a role OA. Studies also demonstrated that inhibition of SMURF1-mediated degradation of Smad1/5 increased BMP-2 signal responsiveness and enhanced osteoblastic activity [42] and that intracellular delivery of SMURF1 inhibited BMP-2-induced alkaline phosphatase activity and downregulated BMP receptors [43, 44]. Remarkably, PC upregulated the expression of TGFRB1, Smad3, and SMURF1 in the OA chondrocytes. This up-regulatory effect indicates that PC may exert its OA disease modifying effect or chondroprotective effects, in part, by enhancing the signaling via the TGFBR1 and Smad2/3 route while suppressing the signaling via IL-7, Smad1/5, and BMP-2 route.

Previous studies showed that inhibition of IGFBP5 proteolysis reduced articular cartilage degeneration [45-47], PDLIM7 inhibited MMP production [48], and that the expression of FGFR2 was decreased in degenerated cartilage [49]. These previous findings indicate that IGFBP5, PDLIM7, and FGFR2 are potentially chondroprotective proteins. In contrast, BMP-2 is potentially a disease candidate gene. The level of BMP-2 was elevated in serum, synovial fluid, and chondrocytes derived from OA patients and BMP2 induced chondrocyte hypertrophy and osteophyte formation [50-52]. Here, we demonstrated that PC up-regulated the expression of IGFBP5, PDLIM7, and FGFR2 while downregulated the expression of BMP2. This differential effect of PC on the expression of these genes indicates that PC may exert its OA disease modifying effect, in part, by stimulating the production of chondroprotective proteins and inhibiting pathological ossification stimulated by BMP2.

Of the genes classified in the inflammatory response, skeletal system development, and wnt receptor signaling pathways, PC downregulated the expression of FABP4, IRAK2, DLX2, POSTN, APCDD1, NDP, SOX4, and FZD8. These genes have either been previously implicated in arthritis or terminal differentiation of chondrocytes. For example, serum FABP4 was increased in patients with rheumatoid arthritis and metabolic syndrome [53, 54]; DLX2 promoted osteogenic differentiation of stem cells and enhanced mineralization [55]; POSTN induced the expression of IL-6, IL-8, and MMPs [56]; APCDD1 was associated with increased expression of RunX2 and elevated alkaline phosphatase activity [57]; NDP activated Wnt/β-catenin pathway associated with OA [58, 59]; SOX-4 signaling resulted in repression of SOX-9, a master transcription factor critical for chondrogenesis [60]; Silence of FZD8 resulted in decreased expression of MMP-3 [61]. The downregulation of the expression of these genes by PC was consistent with its OA disease-modifying activity.

## CONCLUSION

In conclusion, PC can act directly on OA chondrocytes in the absence of calcium crystals. PC may exert its OA disease modifying effect, in part, through a crystal-independent mechanism or by inhibiting the expressions of genes implicated in chondrocyte activation, proliferation, apoptosis, calcification, and inflammatory response while stimulating the expressions of many genes implicated in chondroprotection and matrix production in chondrocytes.

## Figures and Tables

**Fig. (1):**
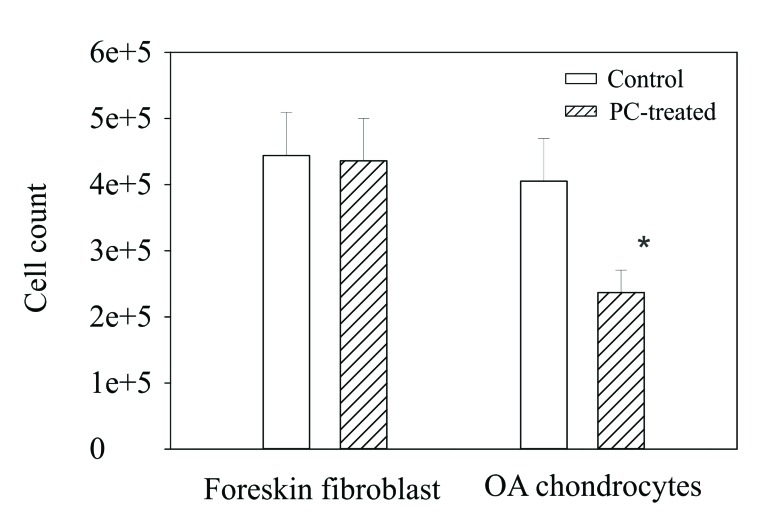
Effect of PC on chondrocyte proliferation. Left bar group-numbers of foreskin fibroblasts cultured in the absence and presence of PC. No effect was noted. Right bar group - numbers of OA chondrocytes cultured in the absence and presence of PC. PC inhibited the proliferation of OA chondrocytes. * = p < 0.01 versus untreated control.

**Fig. (2):**
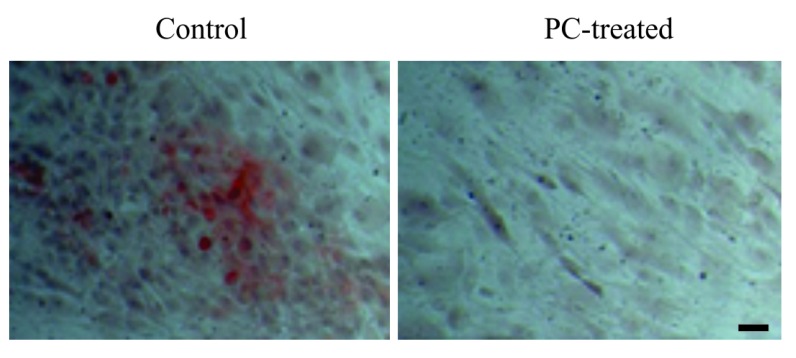
Representative images of alizarin red stained OA chondrocytes. Calcium deposits were detected in monolayer of OA chondrocytes cultured in osteogenetic differentiation medium, but not in the monolayer of OA chondrocytes cultured in osteogenetic differentiation medium containing 1 mm PC. (bar = 10 μm).

**Fig. (3):**
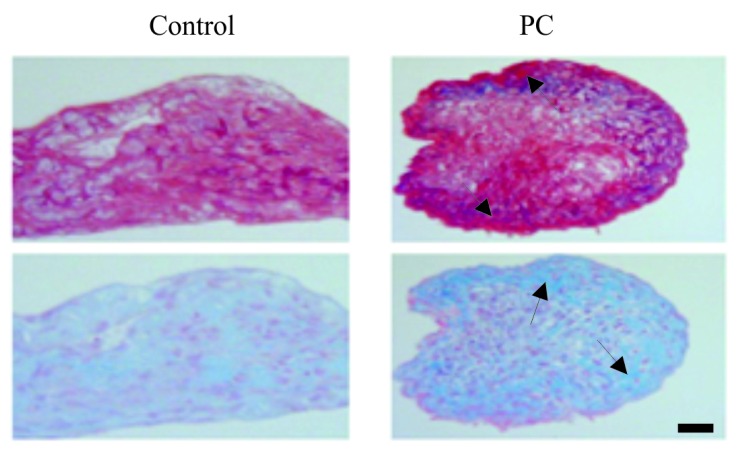
Representative images of picrosirius red and alcian blue stained sections of micromasses of OA chondrocytes. Arrows – outer layers of the micromasses of OA chondrocytes. (bar = 100 μm). PC stimulated the production of collagens and proteoglycans.

**Table 1:** Differentially expressed genes in PC-treated via untreated OA chondrocytes.

**Biological** **process**	**Gene ID**	**Gene** **Identifier**	**Fold** change*	**Description**
Cell proliferation				
	HELLS	NM_018063	-2.32	Helicase, lymphoid-specific
	NDP	NM_000266	-2.30	Norrie disease (pseudoglioma)
	EREG	NM_001432	-2.18	Epiregulin
	KIF15	NM_020242	-1.98	Kinesin family member 15
	SPRY2	NM_005842	-1.92	Sprouty homolog 2 (Drosophila)
	ENPP7	AA701973	-1.91	Ectonucleotide pyrophosphatase/phosphodiesterase 7
	E2F7	AI341146	-1.87	E2F transcription factor 7
	IL7	NM_000880	-1.82	Interleukin 7
	UHRF1	AK025578	-1.78	Ubiquitin-like with PHD and ring finger domains 1
	CDK5R1	AL567411	-1.75	Cyclin-dependent kinase 5, regulatory subunit 1 (p35)
	TACC3	NM_006342	-1.75	Transforming, acidic coiled-coil containing protein 3
	NTN1	BF591483	-1.75	Netrin 1
	EEF1E1	N32257	-1.74	Eukaryotic translation elongation factor 1 epsilon 1
	TCF19	BC002493	-1.74	Transcription factor 19
	GINS1	NM_021067	-1.73	GINS complex subunit 1 (Psf1 homolog)
	NR2F2	AL554245	-1.72	Nuclear receptor subfamily 2, group F, member 2
	CCND1	BC000076	-1.68	Cyclin D1
	SOX4	AI989477	-1.67	SRY (sex determining region Y)-box 4
	MEF2C	N22468	-1.66	Myocyte enhancer factor 2C
	CDKN2D	U20498	-1.63	Cyclin-dependent kinase inhibitor 2D (p19, inhibits CDK4)
	HBEGF	NM_001945	-1.62	Heparin-binding EGF-like growth factor
	FOSL1	BG251266	-1.58	FOS-like antigen 1
	MKI67	AU152107	-1.58	Antigen identified by monoclonal antibody Ki-67
	TRIM24	NM_015905	-1.57	Tripartite motif-containing 24
	SPHK1	NM_021972	-1.56	Sphingosine kinase 1
	BLM	NM_000057	-1.55	Bloom syndrome, RecQ helicase-like
	BMP2	NM_001200	-1.55	Bone morphogenetic protein 2
	ACER3	R12678	-1.55	Alkaline ceramidase 3
	CDCA7	AY029179	-1.52	Cell division cycle associated 7
	CDC20	NM_001255	-1.50	Cell division cycle 20 homolog (S. cerevisiae)
	B4GALT1	M22921	2.20	UDP-Gal:betaGlcNAc beta 1,4- galactosyltransferase, polypeptide 1
	FGFR2	M80634	1.99	Fibroblast growth factor receptor 2
	EGR1	AI459194	1.98	Early growth response 1
	TXNIP	AA812232	1.82	Thioredoxin interacting protein
	TGFBR1	AV700621	1.74	Transforming growth factor, beta receptor 1
	DDR2	AA545764	1.58	Discoidin domain receptor tyrosine kinase 2
	CSF1	U22386	1.55	Colony stimulating factor 1 (macrophage)
	IGFBP5	R73554	1.53	Insulin-like growth factor binding protein 5
	ARIH2	AW074830	1.51	Ariadne homolog 2 (Drosophila)
Apoptosis				
	HELLS	NM_018063	-2.32	Helicase, lymphoid-specific
	DUSP6	BC003143	-2.30	Dual specificity phosphatase 6
	SPRY2	NM_005842	-1.92	Sprouty homolog 2 (Drosophila)
	PCGF2	NM_007144	-1.89	Polycomb group ring finger 2
	IL7	NM_000880	-1.82	Interleukin 7
	BIRC5	AA648913	-1.76	Baculoviral IAP repeat-containing 5
	CDK5R1	AL567411	-1.75	Cyclin-dependent kinase 5, regulatory subunit 1 (p35)
	RASSF5	BC004270	-1.75	Ras association (RalGDS/AF-6) domain family member 5
	NTN1	BF591483	-1.75	Netrin 1
	EEF1E1	N32257	-1.74	Eukaryotic translation elongation factor 1 epsilon 1
	PAK1	AU147145	-1.73	P21 protein (Cdc42/Rac)-activated kinase 1
	BCAP29	AL583687	-1.72	B-cell receptor-associated protein 29
	LMNB1	NM_005573	1.70	Lamin B1
	EPHA2	NM_004431	-1.69	EPH receptor A2
	PHLDA1	AK026181	-1.68	Pleckstrin homology-like domain, family A, member 1
	ALDH1A3	NM_000693	-1.67	Aldehyde dehydrogenase 1 family, member A3
	ALKBH1	AI922200	-1.67	AlkB, alkylation repair homolog 1 (E. coli)
	SOX4	AI989477	-1.67	SRY (sex determining region Y)-box 4
	MEF2C	N22468	-1.66	Myocyte enhancer factor 2C
	RNF130	AL831873	-1.64	Ring finger protein 130
	CDKN2D	U20498	-1.63	Cyclin-dependent kinase inhibitor 2D (p19, inhibits CDK4)
	FOSL1	BG251266	-1.58	FOS-like antigen 1
	TRIM24	NM_015905	-1.57	Tripartite motif-containing 24
	SPHK1	NM_021972	-1.56	Sphingosine kinase 1
	BMP2	NM_001200	-1.55	Bone morphogenetic protein 2
	CDC2	AL524035	-1.54	Cell division cycle 2, G1 to S and G2 to M
	CRADD	U79115	-1.53	CASP2 and RIPK1 domain containing adaptor with death domain
	EXPL1	D79987	-1.53	extra spindle pole bodies homolog 1 (S. cerevisiae)
	PHLDA1	NM_007350	-1.52	Pleckstrin homology-like domain, family A, member 1
	MAP1S	NM_018174	-1.52	Microtubule-associated protein 1S
	MCL1	BF594446	2.33	Myeloid cell leukemia sequence 1 (BCL2-related)
	B4GALT1	M22921	2.20	UDP-Gal:betaGlcNAc beta 1,4- galactosyltransferase, polypeptide 1
	MX1	NM_002462	2.02	Myxovirus resistance 1, interferon-inducible protein p78 (mouse)
	FGFR2	M80634	1.99	Fibroblast growth factor receptor 2
	TXNIP	AA812232	1.82	Thioredoxin interacting protein
	TGFBR1	AV700621	1.74	Transforming growth factor, beta receptor 1
	ARRB2	NM_004313	1.61	Arrestin, beta 2
	SMAD3	BF971416	1.55	SMAD family member 3
TGF-β receptor signaling pathway				
	TGFBR1	AV700621	1.74	Transforming growth factor, beta receptor 1
	ARRB2	NM_004313	1.61	Arrestin, beta 2
	C5orf13	U36189	1.59	Chromosome 5 open reading frame 13
	SMURF1	AC004893	1.57	SMAD specific E3 ubiquitin protein ligase 1
	SMAD3	BF971416	1.55	SMAD family member 3
Ossification				
	FGFR2	M80634	1.99	Fibroblast growth factor receptor 2
	RSAD2	AW189843	1.63	Radical S-adenosyl methionine domain containing 2
	PDLIM7	AW206786	1.57	PDZ and LIM domain 7 (enigma)
	SMURF1	AC004893	1.57	SMAD specific E3 ubiquitin protein ligase 1
	IGFBP5	R73554	1.53	Insulin-like growth factor binding protein 5
	ENPP1	BF057080	-1.66	Ectonucleotide pyrophosphatase/phosphodiesterase 1
	BMP2	NM_001200	-1.55	Bone morphogenetic protein 2

*Negative number indicates decreased expression and positive number indicates increased expression (fold change) in PC-treated OA chondrocytes compared with untreated OA chondrocytes.

**Table 2:** Differentially expressed genes in PC-treated via untreated OA chondrocytes.

	**Gene ID**	**Gene** **Identifier**	**Fold** change*	**Description**
Inflammatory response				
	FABP4	NM_001442	-2.11	Fatty acid binding protein 4, adipocyte
	IRAK2	AI246590	-1.71	Interleukin-1 receptor-associated kinase 2
	IL1RAP	AF167343	-1.57	Interleukin 1 receptor accessory protein
	BMP2	NM_001200	-1.55	Bone morphogenetic protein 2
	B4GALT1	M22921	2.20	UDP-Gal:betaGlcNAc beta 1,4- galactosyltransferase, polypeptide 1
	FN1	AJ276395	1.92	Fibronectin 1
Skeletal system development				
	DLX2	NM_004405	-2.37	Distal-less homeobox 2
	PCGF2	NM_007144	-1.89	Polycomb group ring finger 2
	EPHA2	NM_004431	-1.69	EPH receptor A2
	POSTN	AW137148	-1.56	Periostin, osteoblast specific factor
	BMP2	NM_001200	-1.55	Bone morphogenetic protein 2
	FGFR2	M80634	1.99	Fibroblast growth factor receptor 2
	TGFBR1	AV700621	1.74	Transforming growth factor, beta receptor 1
	RDH10	NM_172037	1.62	Retinol dehydrogenase 10 (all-trans)
Wnt receptor signaling pathways				
	APCDD1	N48299	-2.68	Adenomatosis polyposis coli down-regulated 1
	NDP	NM_000266	-2.30	Norrie disease (pseudoglioma)
	CCND1	BC000076	-1.68	Cyclin D1
	SOX4	AI989477	-1.67	SRY (sex determining region Y)-box 4
	FZD8	AB043703	-1.58	Frizzled homolog 8 (Drosophila)
	BMP2	NM_001200	-1.55	Bone morphogenetic protein 2
	FGFR2	M80634	1.99	Fibroblast growth factor receptor 2
	SMAD3	BF971416	1.55	SMAD family member 3

*Negative number indicates decreased expression and positive number indicates increased expression (fold change) in the PC-treated OA chondrocyte compared with the untreated OA chondrocytes.

**Table 3:** Differential expression confirmed by real-time RT-PCR.

**Gene ID**	**Gene Identifier**	**Differential Expression** **Microarray**	**Differential Expression** **RT-PCR**
HELLS	NM_018063	-2.32	-2.97
NDP	NM_000266	-2.30	-2.01
CCND1	BC000076	-1.68	-2.03
IL7	NM_000880	-1.82	-1.75
ENPP1	BF057080	-1.66	-1.92
FABP4	NM_001442	-2.11	-1.81
THBD	NM_000361	-2.57	-2.04
PLAT	NM_000930	-2.34	-2.50
FGFR2	M80634	1.99	2.34
SMAD3	BF971416	1.55	1.45
IGFBP5	R73554	1.53	1.67
